# ‘Natural experiment’ Demonstrates Top-Down Control of Spiders by Birds on a Landscape Level

**DOI:** 10.1371/journal.pone.0043446

**Published:** 2012-09-07

**Authors:** Haldre Rogers, Janneke Hille Ris Lambers, Ross Miller, Joshua J. Tewksbury

**Affiliations:** 1 Department of Biology, University of Washington, Seattle, Washington, United States of America; 2 University of Guam, Western Pacific Tropical Research Center, Mangilao, Guam; University of San Diego, United States of America

## Abstract

The combination of small-scale manipulative experiments and large-scale natural experiments provides a powerful approach for demonstrating the importance of top-down trophic control on the ecosystem scale. The most compelling natural experiments have come from studies examining the landscape-scale loss of apex predators like sea otters, wolves, fish and land crabs. Birds are dominant apex predators in terrestrial systems around the world, yet all studies on their role as predators have come from small-scale experiments; the top-down impact of bird loss on their arthropod prey has yet to be examined at a landscape scale. Here, we use a unique natural experiment, the extirpation of insectivorous birds from nearly all forests on the island of Guam by the invasive brown tree snake, to produce the first assessment of the impacts of bird loss on their prey. We focused on spiders because experimental studies showed a consistent top-down effect of birds on spiders. We conducted spider web surveys in native forest on Guam and three nearby islands with healthy bird populations. Spider web densities on the island of Guam were 40 times greater than densities on islands with birds during the wet season, and 2.3 times greater during the dry season. These results confirm the general trend from manipulative experiments conducted in other systems however, the effect size was much greater in this natural experiment than in most manipulative experiments. In addition, bird loss appears to have removed the seasonality of spider webs and led to larger webs in at least one spider species in the forests of Guam than on nearby islands with birds. We discuss several possible mechanisms for the observed changes. Overall, our results suggest that effect sizes from smaller-scale experimental studies may significantly underestimate the impact of bird loss on spider density as demonstrated by this large-scale natural experiment.

## Introduction

After a 40+ year focus on manipulative experiments in ecology, there has been a recent resurgence in observational approaches, often through ‘natural experiments’ resulting from large-scale unintentional perturbations to natural systems [1]. While manipulative experiments offer powerful approaches for elucidating ecological mechanisms [1], [2], they may be less useful at accurately assessing cascading impacts and compounding effect sizes because manipulations are often only possible at scales far smaller than the processes under study [1], [3]. Natural experiments, on the other hand, offer only a limited window into mechanism, but can provide novel insights about the potential effect size associated with a perturbation, as they incorporate the capacity of the entire system to shift in response to the treatment. In this way, natural experiments can serve to validate controlled experimental findings across a heterogeneous landscape, a critical step if research is to be used for management purposes. Our most complete understanding of many basic ecological questions has come from the complementary approaches of small-scale planned experiments and large-scale ‘natural experiments’ [1]; the series of inter-island comparisons and experiments manipulating lizard and spider populations in the Bahamas provide a classic example of this approach [4]–[6].

A combination of natural experiments and manipulative experiments has been important in the demonstration of top-down control of lower trophic levels by apex predators [4], a process that often occurs on a large spatial and long temporal scale [7]. The systematic loss of top predators – a process recently referred to as trophic downgrading – is argued to be among humankind's most pervasive influence on nature, creating cascading impacts that change a wide range of ecosystem dynamics [8]. Natural experiments based on apex predator loss have provided convincing examples of the importance of top predators across a landscape, while manipulative experiments have been useful in developing predictions about the conditions under which top-down control occurs [7], [8]. Classic examples of natural experiments can be found in many different ecosystems across the globe, from sea otters in Pacific kelp forests, to wolves in Yellowstone, to land crabs on Christmas Island, to fish overharvesting in Jamaican coral reefs [9]–[12]. However, with the seeming ubiquity of top-down control, it is striking that there is currently no landscape-level natural experiment that demonstrates the role of insectivorous birds, one of the most well studied and widespread groups of top predators in the world. Whelan, Wenny and Marquis [13] identified this knowledge gap, and urged “ecologists to be poised to take advantage of “natural” experiments that may arise, for instance, from geographically local declines of certain species or groups of species or from epi-zootics like that of West Nile Virus”. In the meantime, we rely on results from small-scale manipulations to understand the ecosystem function provided by insectivorous birds, and ultimately, to predict the impact of the ongoing and future insectivorous bird decline across the landscape [14], [15].

The most consistent result from manipulative experiments testing the impact of birds on arthropods is an increase in spiders, although experimental limitations limit our ability to extrapolate these results to the large scales on which they operate [13], [16]. A recent review of 36 experiments that used experimental bird exclosures to determine the impact of birds on spiders found significant increases in spider densities inside bird exclosures relative to control areas in 75% of the studies [16]. However, exclosure experiments suffer from restricted spatial scales and temporal durations, as well as possible exclosure effects, which collectively have potential to either enhance or muffle the true ecological response. Most exclosures cover a single branch; the largest in the aforementioned review [16] was 4×4 meters [17]; the small spatial scale allows migration into and out of exclosures by both spiders and their prey. The duration of studies was short, ranging from 26 days to 33 months, with an average duration of 10 months and a median of 6 months [16]. These limited spatial and temporal scales make it very difficult to assess the full demographic response that might be seen in spider communities following the removal of birds. Finally, the exclosures themselves are typically built from bird netting, which provides attachment points for spiders and is rarely controlled for in these experiments [16]. This increase in attachment points would artificially inflate the spider abundance in the exclosure area. While the increase of spiders is relatively consistent across studies, there are many sources of variation from the manipulative experimental design that might affect this relationship and the overall effect size.

The most direct mechanism linking bird presence to spider population size is predation by birds, however, there are alternative mechanisms that could lead to an increase in spiders when birds are excluded. Bird consumption of spiders is considered intraguild predation since both consume herbivorous arthropods. Therefore, the intermediate predators, spiders, may be released from competition for prey in the absence of the top predators; in a related system, spider populations were higher on islands without lizards (predators of spiders) than on islands with lizards due to release from competition in addition to release from direct predation [18]. In addition, birds may cause behavioral responses in spiders that limit spider populations, as has been shown in systems with spiders as predators of grasshoppers [19] or wolves as predators of elk [20]. The review by Gunnarsson [16] and most bird exclosure studies focus on direct predation by birds, leaving alternative mechanisms relatively unexplored.

**Figure 1:**
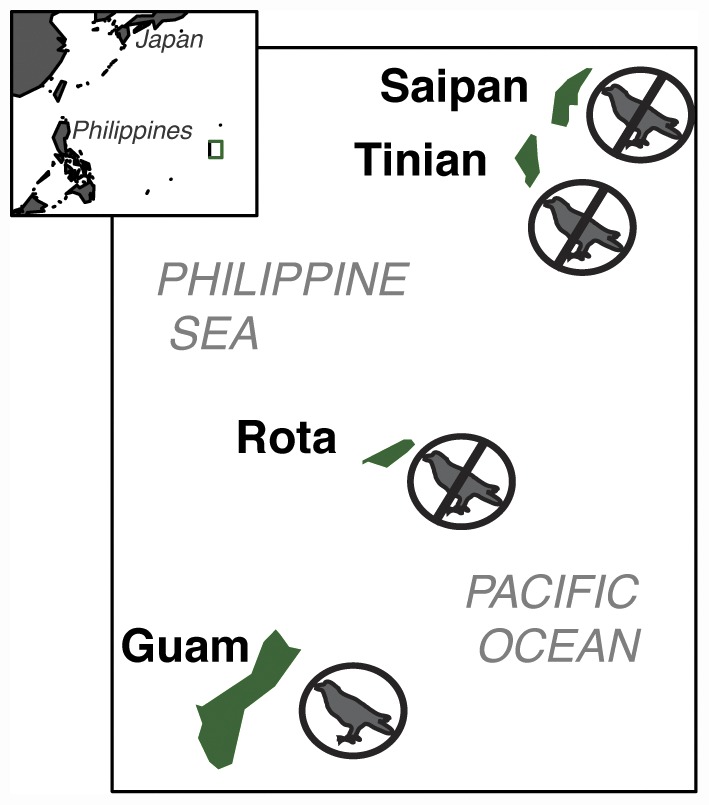
Map of the Mariana Islands. All forest birds are functionally extinct on the island of Guam, whereas relatively healthy bird populations remain on three nearby islands of Saipan, Tinian and Rota.

Here we present the first landscape level natural experiment, to our knowledge, of the impacts of bird loss on top-down control of their prey, spiders. Our objective is to compare the directionality and magnitude of effects generated from long-term, landscape-scale bird loss to the effects generated from bird exclusion experiments elsewhere. To do this, we take advantage of the only place in the world where all avian insectivores have been functionally extirpated from the landscape, the Western Pacific island of Guam. The brown tree snake (*Boiga irregularis*) was introduced to Guam (Figure 1) in the mid-1940's, leading to the extirpation of all native insectivorous bird species from the majority of the island in the mid-1980's [21]–[23]. There are two insectivorous bird species remaining today, in extremely localized populations; the Micronesian Starling (*Aplonis opaca*) has a small population on Andersen Air Force Base at the northern tip of Guam and the Mariana Swiftlet (*Aerodramus bartschi*) inhabits three caves on the Naval Base in southern Guam. No non-native insectivorous bird species have established in the forests of Guam, therefore, aside from these two locations on the military bases, the forests are devoid of insectivorous birds.

Since no spider surveys of which we are aware exist from prior to the loss of birds, we used a space-for-time approach, comparing the abundance of spider webs along transects in native forest on Guam to that on three nearby islands (Rota, Tinian and Saipan; Figure 1) that have no known snake populations and similar bird communities to Guam before the snake invasion [24]. Thus the scale of our comparison is between 4 islands, one of which has been without top-down control by birds for the past 25+ years. We also compare spider web size between islands for one common spider species, *Argiope appensa*, to shed light on potential mechanisms for differences in spider populations between islands.

## Materials and Methods

### Ethics Statement

All necessary permits were obtained for the described field studies. Permits or permission for the use of these sites were obtained from the Government of Guam Forestry Division (for all Guam sites), the Commonwealth of the Northern Mariana Islands Division of Fish and Wildlife (for all sites on Saipan and Rota, and some sites on Tinian), and from the US Navy (NAVFACMAR office, for some sites on Tinian). The field studies did not involve endangered or protected species.

### Site Description

The study was conducted on the Micronesian islands of Guam, Rota, Tinian and Saipan (Figure 1). These four islands are the southernmost inhabited islands of the Mariana Island chain. All four islands have an average annual temperature around 27 degrees C and little seasonal temperature fluctuation. Guam averages 2,586 mm of rainfall per year, whereas Saipan receives 1900–2300 mm of precipitation per year, depending on location [25], [26]. Rota and Tinian are intermediate between Guam and Saipan in terms of temperature and precipitation.

The primary forest type on Guam, Rota, Tinian and Saipan grows on a rugged limestone karst substrate; there are approximately 40 tree and shrub species in this forest type (Rogers, pers. obs.). We selected 4–8 comparable native limestone karst forest areas on each of the four islands, henceforth called sites. These sites contained similar tree species; they were dominated by some combination of *Aglaia mariannensis*, *Guamia mariana*, *Cynometra ramiflora*, *Psychotria mariana*, and *Eugenia reinwardtiana*, and contained large individuals of *Pisonia grandis*, *Ficus* spp., and/or *Premna obtusifolia*. Due to widespread activity during WWII and several thousand years of human occupation, the forests on all four islands have had some disturbance leading to degradation of the karst substrate. However, all chosen sites had some karst substrate remaining; the more disturbed sites had scattered karst rocks on top of soil and the least disturbed sites had intense karst substrate with small pockets of soil.

### Insectivorous birds

The bird communities are similar between islands; there is either the same species or a functional overlap for nearly all species (e.g. both a white-eye species and a kingfisher species are present on each island, although the same species is not present on all four islands). Prior to the introduction of the brown tree snake, ten insectivorous bird species were present in the forests of Guam: Bridled White-eye (*Zosterops conspicillatus*), Rufous Fantail (*Rhipidura rufifrons*), Micronesian Starling (*Aplonis opaca*), Mariana Swiftlet (*Aerodramus bartschi*), Cardinal Honeyeater (*Myzomela rubratra*), Nightingale Reed-Warbler (*Acrocephalus luscinia)*, Mariana Crow (*Corvus kubaryi*), Micronesian Kingfisher (*Todiramphus cinnamominus*), Guam Flycatcher (*Myiagra freycineti*), and the Guam Rail (*Gallirallus owstoni*) (Table 1). While there has been little work on the diet of Mariana birds, many of these species have been observed eating spiders and/or using webs as nest material (Table 1).

**Table 1:** Distribution of invertebrate-feeding birds in the Mariana Islands, and observations of spider predation and use of spider webs by birds.^1^

Species	Guam	Rota	Tinian	Saipan	Spiders in diet?	Webs as nest material	Source of spider observations
Mariana Swiftlet (*Aerodramus bartschi*)	**P**	**HP**	**HP**	**P**	No	No	[44], [45]
Micronesian Starling (*Aplonis opaca*)	**P**	**P**	**P**	**P**	No data	Unlikely	[46]
Nightingale Reed Warbler (*Acrocephalus luscinia*)	**HP**	**P**	**P**	**P**	**Yes**	**Yes**	[47], [48]
Micronesian Megapode (*Megapodius laperouse*)	**HP**	**HP**	**HP**	**P**	**Yes**	No data	[49]
Cardinal Honeyeater (*Myzomela rubrata saffordi*)	**HP**	**P**	**P**	**P**	No data	**Yes**	[46]
Rufous Fantail (*Rhipidura rufifrons*)	**HP**	**P**	**P**	**P**	**Yes**	**Yes**	[46], P. Luscomb & H. Roberts, pers.comm.
Bridled White-eye (*Zosterops conspicillatus*)	**HP**	A	**P**	**P**	No data	**Yes**	P. Radley & H. Roberts, pers.comm.
Mariana Crow (*Corvus kubaryi*)	**HP**	**P**	A	A	**Yes**	No data	L. Berry, pers.comm.
Guam Rail (*Gallirallus owstoni*)	**HP**	I	A	A	No data	No data	
Micronesian Kingfisher (*Halcyon c. cinnamomina*)	**HP**	A	A	A	No data	No	[50]
Guam Flycatcher (*Myiagra freycineti*)	**HP**	A	A	A	No data	**Yes**	[46]
Collared Kingfisher (*Todiramphus chloris*)	A	**P**	**P**	**P**	**Yes**	No	[47]
Golden White-eye (*Cleptornis marchei*)	A	A	**HP**	**P**	**Yes**	**Yes**	[51], H. Roberts, pers.comm.
Rota Bridled White-eye (*Zosterops rotensis*)	A	**P**	A	A	No data	**Yes**	[52]
Tinian Monarch (*Monarcha takatsukasae*)	A	A	**P**	A	**Yes**	**Yes**	P. Luscomb & P. Radley, pers.comm.

1 P = currently present, HP = historically present but now extinct in the wild, A = absent, I = introduced for conservation purposes.

Only two of the 10 native insectivorous forest bird species remain on Guam: the Micronesian Starling (*Aplonis opaca*), and the Marianas Swiftlet (*Acrocephalus luscinia*). *Aplonis opaca* has a localized remnant population of less than 400 birds (D. Vice, J. Quitagua and L. Obra, pers.comm.) covering an area of less than 50 km^2^, and *Acrocephalus luscinia* has remnant populations numbering around 1100 in three caves in Southern Guam (A. Brooke, pers.comm.) covering an area of around 12 km^2^. Both regions hosting remnant bird populations on Guam are on military bases in areas heavily trapped for snakes. Swiftlets were never observed at our field sites, which are in the northern half of the island. We have observed 1–2 juvenile starlings intermittently in the general area of the field site closest to Andersen Air Force Base; when present, these birds are hard to miss because the forest is otherwise silent. However we do not believe these individual birds could have a significant impact on spiders in the large section of forest they inhabit. Starlings have not been observed at our other sites in the last 7 years of field research.

### Spider Surveys

We compared the abundance of web-building spiders on Guam to that on Rota, Tinian and Saipan. At each site, we set up 1–3 transects, separated by at least 200 meters. The transects were 20 or 30 meters long, depending on the year. We counted all visible webs within 1 horizontal meter of each transect centerline and up to 2 vertical meters above the ground. Webs lacking a spider were considered abandoned, and not counted. Webs were categorized as “orb”, “tent” or “miscellaneous” web type. Webs from all three categories were used in the comparison of total number of webs, although the qualitative results did not change for each category alone. We identified individual spiders to species if they belonged to either of the most common and identifiable species, *Argiope appensa* and *Cyrtophora mollucensis*, and measured the area of *Argiope appensa* webs by taking two diameter measurements 90 degrees apart (height and width).

We conducted surveys in the wet season from July to September 2007, and in the dry season from April to May 2008, to capture seasonal impacts of bird loss on spiders. A total of 70 transects were surveyed, 31 transects in the dry season (Guam, n = 8; Rota, n = 7; Tinian, n = 21; Saipan, n = 25) and 39 transects in the wet season (Guam, n = 8; Rota, n = 1, Tinian, n = 13, Saipan, n = 17).

### Analysis

Our analysis tested 1) whether bird presence was a significant predictor of spider web abundance and 2) whether the impact of bird presence differed by season. All data were analyzed using a linear mixed effects model with a Poisson error distribution. The number of webs per transect was the response variable, bird presence (yes/no), season (wet/dry), and bird presence∶season interaction were fixed effects; site was a random effect. We did not include transect length as a fixed effect because season correlated completely with length (wet season transects were 30 m long, dry season transects were 20 m long). We also compared models with length as a fixed effect instead of season, with qualitatively the same results. We identified the best fitting model with Akaike's Information Criterion (AIC) values [27]. To test whether bird presence was a significant predictor for the number of spider webs, we used a likelihood ratio test comparing a full model (bird*season) with a null model having only season as a fixed effect. To test whether the impact of birds differed between wet and dry season, we compared a model including a bird presence∶season interaction term to a model without this term, also using a likelihood ratio test.

We compare our effect sizes to those reported in Gunnarsson's review of the impact of birds on spider populations [16]. We focus on the subset of the studies that were conducted in the tropics, as these are likely to be more relevant to our own study, although the inclusion of temperate studies does not qualitatively change the pattern. As in Gunnarsson [16] and Gruner [17], we chose to exclude *Achaearanea* cf. *riparia*, the invasive spider species whose irruption in the absence of birds was enhanced by high abundances of juvenile spiders.

We also tested whether bird presence affected web size of the common spider, *Argiope appensa*. We combined data from the two survey rounds, as few webs were found during the 2007 surveys on islands with birds. An analysis of the 2008 data alone produced the same results as an analysis including data from both years. To test whether the size of *Argiope appensa* webs is related to the presence of birds, we used a linear mixed effects model with the log of web area as the response and site as a random effect. We identified the best fitting model with AIC values [27]. To test for the significance of birds, we used a likelihood ratio test comparing a model with bird presence (yes/no) as a main effect to a model without a main effect (intercept only null model); site was included as a random effect in both models.

All data analysis was performed using R v2.13.0 [28], using the lme4 package (version 0.999375-39, published 3 Mar 2011).

## Results

**Figure 2:**
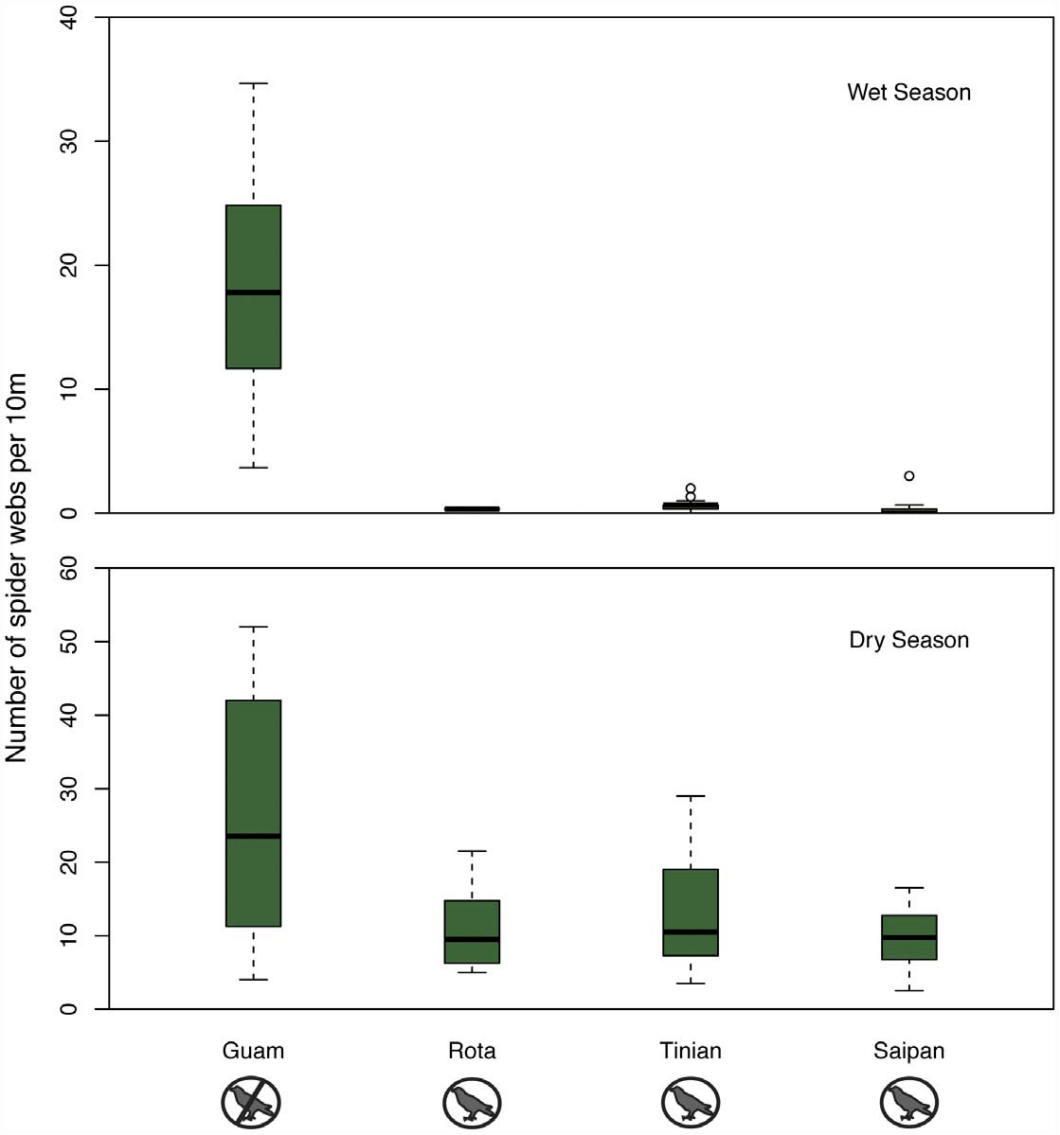
Forest spider web abundance by island. The bar in each boxplot indicates the median number of webs per 10 meters, while the box shows the first and third quartiles of data. The whiskers range from the lowest to the highest data points within 1.5 * the interquartile range of the lower and upper quartiles, respectively. Any data points beyond the range of the whiskers are considered outliers and plotted with a circle. Statistical models indicate that webs are less common on islands with birds (Rota, Tinian, Saipan) than on Guam (no birds) in both the a) wet and b) dry season.

Guam, without birds, had a mean of 18.37 spider webs per ten meters in the wet season, compared to 0.45 webs per ten meters on nearby islands with birds (Figure 2, Table 2). In the dry season, Guam had 26.19 spider webs per ten meters compared to 11.37 webs per ten meters on nearby islands with birds (Figure 2, Table 2). Thus the ratio of spider webs on islands with birds to Guam, without birds, was 1∶40.8 in the wet season and 1∶2.3 in the dry season. A generalized linear mixed effects model with bird presence (yes/no), season (wet/dry), and bird∶season interaction as predictors fit significantly better than a model without bird presence or the bird∶season interaction, suggesting that bird presence explains web densities (LRT, p<0.001; Table 3), and that effects of birds on web densities differs by season (LRT p<0.001; Table 3). These results not only show dramatic changes in abundance, they also point to the almost complete loss of seasonality in web-building behavior (Figure 2). On islands with birds, the ratio of webs in the dry season to the wet season is 25∶1, but on Guam, the ratio is just 1.4∶1; web densities remain high all year long.

**Table 2:** Summary data from spider surveys.

Island	Season	Number of Transects	Total Distance Surveyed (m)	Total Number of Webs	Number of Webs/10 m
Guam	dry	8	160	419	26.2
	wet	8	233.2	430	18.4
Rota	dry	7	140	156	11.1
	wet	1	30	1	0.3
Saipan	dry	8	160	155	9.7
	wet	17	510	17	0.3
Tinian	dry	8	160	212	13.3
	wet	13	390	24	0.6

**Table 3:** Model selection testing impact of bird presence, season, and their interaction on web abundance.

Model	Log-likelihood	AIC	deltaAIC
Bird presence*season	−152.00	314.00	0
Bird presence+season	−237.74	483.48	169.48
Season	−242.69	491.31	177.31

Site was included as a random effect in all models.

**Figure 3:**
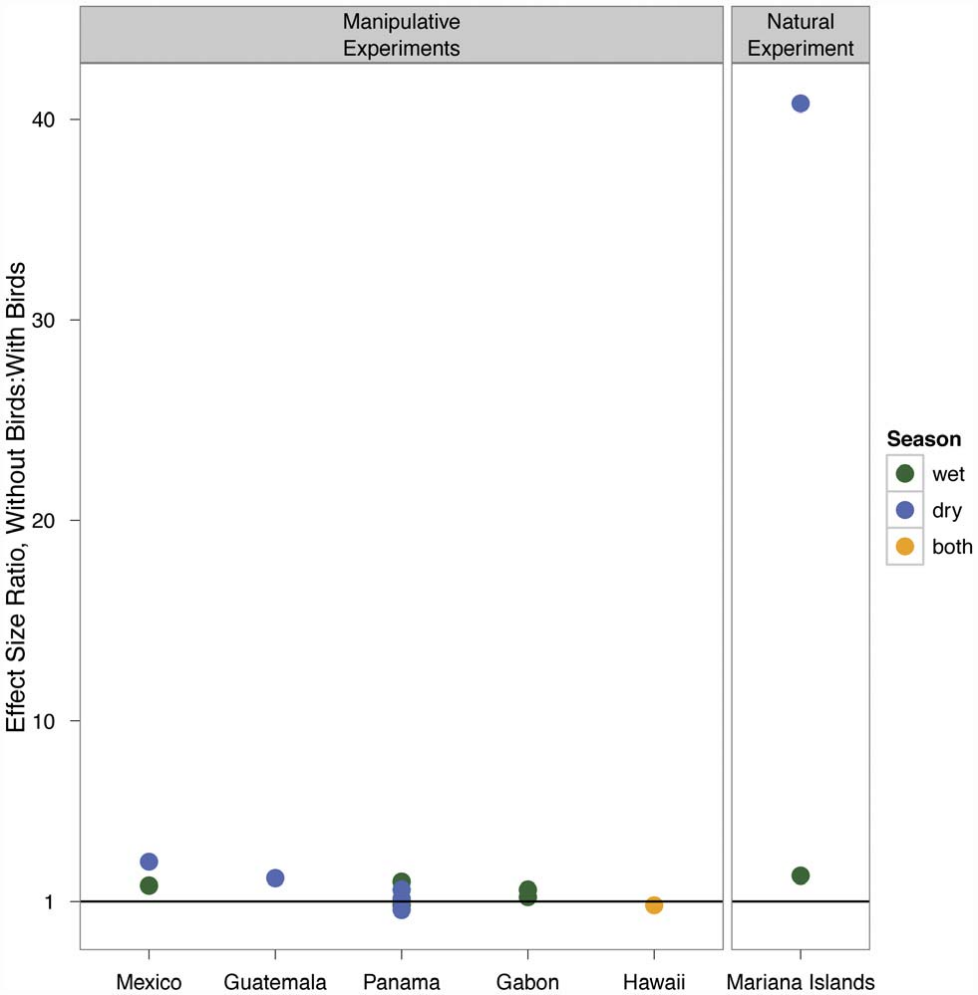
Difference in effect size between manipulative and natural experiments. The effect sizes from manipulative experiments in the tropics included in a recent review paper [16] range from no effect to a 3-fold effect, much less than the effect seen in the dry season during the natural experiment in the Mariana Islands.

We used reported effect sizes from Gunnarsson [16] to compare to the effect size seen in this experiment. The range of effect sizes (ratio of density of spiders in experimental units to control units) reported was from 0.6 to 8.93, with the mean effect size of 2.19 and a median effect size of 1.64. Of experiments conducted in the tropics, the range was from 0.6 to 3, with an average effect size of 1.4, and a median effect size of 1.19 (Figure 3). There was no consistent trend between effect sizes in the wet and dry seasons (Figure 3). The effect size observed in the Mariana Islands during the dry season was within the range of effect sizes seen in other studies, whereas the effect size in the wet season was 4.6 times higher than the largest community response from all studies reported in Gunnarsson [17] and 13.6 times higher than the largest community response among studies in tropical forests (Figure 3). One study excluded by Gunnarsson because the response was due to a single invasive spider species, showed a 25-fold increase the density of that species when birds were excluded, but the rest of the spider community showed no effect of bird exclusion [17], [29].

The two most apparent web-building spider species found in surveys on Guam were an orb-weaver, *Argiope appensa*, and a sheet-web builder, *Cyrtophora mollucensis*. These two relatively large-bodied species are present on all islands, but were not present in surveys on every island because they are in low abundance on islands with birds. *Argiope appensa* webs were 50% larger on Guam than on the other islands (mean area+/−1 SD: islands with birds = 0.04+/−0.09 m^2^, n = 81; Guam = 0.08+/−0.11 m^2^, n = 136); a likelihood ratio test showed that the model including bird presence as a main effect fit significantly better than the model with no main effect (p<0.01,Table 4). We did not sample enough *Cyrtophora mollucensis* on islands with birds for a similar comparison, as only two *Cyrtophora* webs were found along transects on islands with birds, compared to 21 on Guam.

**Table 4:** Model selection testing impact of birds on web area.

Model	Log-likelihood	AIC	deltaAIC
Bird presence	−346.89	701.78	0
Null (intercept only)	−350.98	707.96	6.18

Site was included as a random effect in all models.

## Discussion

The extirpation of birds from Guam over 30 years ago likely led to an explosion of spider populations (Figure 2). This corroborates the results from bird exclosure experiments elsewhere, which found top down control of spiders by birds [16]. However, our results suggest that the impact of bird loss on spider densities could be substantially larger than that estimated from smaller scale experimental studies (Figure 3). The response we observed in the wet season was over 13 times greater than the largest community response recorded in tropical experimental bird exclusion experiments included in the review by Gunnarsson [16]. Several factors could explain the difference in effect size between the manipulative experiments and our natural experiment. First, the shorter time-scales of manipulative exclusion experiments likely prevent a full assessment of equilibrium densities of spiders or spider prey species following bird removal. Although our study cannot determine whether spider populations on Guam have reached an equilibrium following the loss of birds, the timescale is much longer than any exclosure experiment of which we are aware (30+ years compared to 3 years [30]), which may account for the greater effect size. In addition, the small spatial scale of exclosures allows them to be influenced by migration – both of spiders and prey – in and out of the exclosure. Prey emigration from bird exclosures, in particular, may create lower spider densities inside exclosures. Immigration of spiders to the island of Guam from nearby islands and emigration of prey from the island is not likely, due to the vast distances between islands (>58 kilometers over open ocean).

As in previous studies, we found that differences in spider abundances with bird exclusion were context-dependent [16], [17], [31], [32]. There were marked differences between surveys conducted in two different seasons (Figure 2), likely due to seasonal variation in spider abundances, as has been found in other tropical forests [33], [34]. Spider web density was higher in the dry season than the wet season for all islands, and nearly absent in the wet season on the three islands with intact avifauna. Given that spider abundances on Guam were only slightly higher in the dry season than the wet season, even though the rainfall differences between Guam and nearby islands are slight, it is possible that predators control the annual abundance of spiders more than rainfall. Additional surveys with coincident weather data would be useful to determine whether this seasonal pattern is consistent, and whether it is related to rainfall or predators. The phenological shift resulting in a higher prevalence of spider webs in the dry season may alter food web dynamics in this system.

What caused the much greater abundance of spiders on Guam? As with other studies, we assume that release from predation plays a large role [16]. Unfortunately, there have not been extensive diet studies of most of the bird species in the Marianas, so our evidence for predation is primarily from observations. At least four of the bird species extirpated from Guam (Rufous Fantail [*Rhipidura rufifrons*], Mariana Crow [*Corvus kubaryi*], Micronesian Megapode [*Megapodius laperouse*], and Nightingale Reed-Warbler [*Acrocephalus luscinia*), along with two bird species found on Saipan, Tinian and/or Rota (Golden White-eye [*Cleptornis marchei*] and Collared Kingfisher [*Todiramphus chloris*]), each with a closely related species found on Guam, have been seen consuming spiders (Table 1). It is likely that the Micronesian Starling (*Aplonis opaca*) and the Guam Rail (*Gallirallus owstoni*) also eat spiders, based on the generality of their diets, however there is not a record of this in the literature.

Predation may not be the only mechanism for the increase in spider abundance. One of the benefits of natural experiments, as identified by Hewitt et al [2], is the opportunity to make natural history observations, which can inform the broader understanding of a system. By moving between an island without birds and three islands with birds and surveying spider populations in all places, we developed several alternative hypotheses that might also contribute to the increase in spiders on Guam. First, spiders may have been released from competition for shared prey [18]. Second, spiders on Guam may spend less energy re-building webs destroyed by birds flying through them; creating stabilimenta, or prominent silk markings on their webs which are thought to warn off birds; and manufacturing protein-rich silk taken by birds for use as nesting material [35], [36](Table 1). In theory, they could then divert this conserved energy towards increased reproduction. Finally, spiders may respond to the lack of predation by changing their web-building behavior, as has been shown in *Argiope versicolor*, which build larger webs in the absence of a predator than in its presence [37]. This is consistent with our data, which shows that *Argiope appensa* webs are significantly larger on Guam than on the three islands with birds. Larger webs would be an advantage, as they enable increased prey capture [38]. Likely, the increase in spiders on Guam arises from some combination of these mechanisms.

Whether the loss or exclusion of birds consistently leads to a trophic cascade that affects primary producers is still debatable [39], [40]. To date, this has been addressed solely using experimental bird exclosures, which have not shown consistent results with regards to the impact on plants. A recent meta-analysis of 29 studies shows an overall positive impact of birds on plants, through a negative impact on herbivores, but with many exceptions [40]. For example, birds reduced hunting spiders but not web-spinning spiders in an exclosure experiment in Colorado [31]. Other experiments have shown responses to bird exclusion in the canopy, but not the understory [32], [41]. This variation in response could be due to differences in the complexity and redundancy of food webs. In the Marianas, we demonstrated a strong direct link between birds and carnivorous arthropods. Since both birds and spiders prey on herbivorous insects, spider increases may form a partial buffer against the impacts of bird loss on plants. This could reduce the impact of insectivorous bird loss on ecosystem function. Future comparative and experimental exclosure studies in Guam, Saipan, Tinian and Rota linking bird presence across the landscape to intermediate predator abundance (e.g. spiders), herbivore abundance and the growth and survival of plants would provide a strong test of the importance of birds as top predators.

The perennial drawback associated with natural experiments is a lack of replication [2]; our study system is no exception. The effects of bird loss on Guam can only be assessed on Guam, and thus even assessments that use space-for-time substitution techniques are limited in their predictive power off the island of Guam. Even still, by examining the impact of bird loss on spiders in a natural experiment, we were able to sidestep the spatial and temporal limitations of manipulative experiments, and demonstrate that results from small-scale studies focused on the effect of birds on spiders do scale up to the landscape level. Additional landscape-level studies such as ours, potentially paired with experimental exclosures and greater taxonomic resolution, will provide a more comprehensive understanding of the specific mechanisms by which birds exert top-down control on spiders.

Surprisingly, this natural experiment on the island of Guam has been going on for more than 25 years, yet this is the first study that shows the trophic impact of this bird loss. As ecologists, we need to capitalize on opportunities like this, as they provide information that is complementary to the many manipulative experiments investigating the role of birds, and at an ecologically relevant scale. As bird populations decline around the world [42], [43], understanding the ecological role of insectivorous birds within forest systems is critical. If insectivorous birds continue to decline, we will likely be living in a more spider-dominant world in the future.
