# Correction: ‘Natural experiment’ Demonstrates Top-Down Control of Spiders by Birds on a Landscape Level

**DOI:** 10.1371/annotation/b294c406-c8ae-4c89-a083-5e6e26fb8f22

**Published:** 2013-04-10

**Authors:** Haldre Rogers, Janneke Hille Ris Lambers, Ross Miller, Joshua J. Tewksbury

There were errors in Figure 1 and Figure 3. The correct Figure 1 can be viewed here: 

**Figure:**
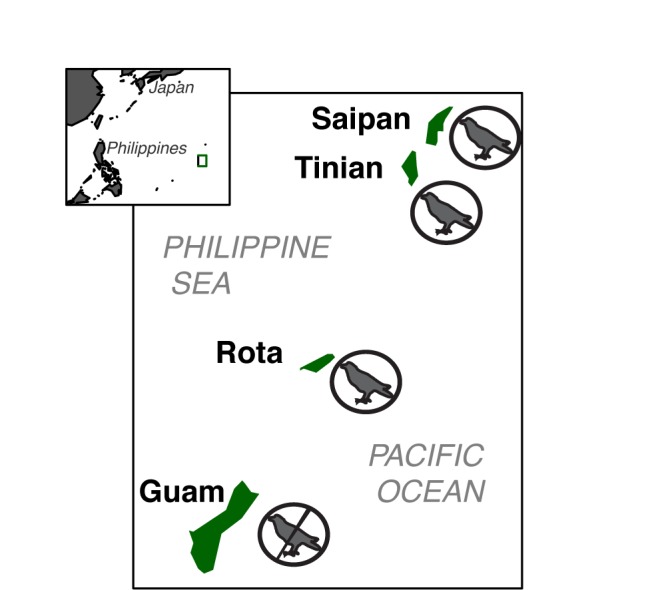


. The correct Figure 3 can be viewed here: 

**Figure:**
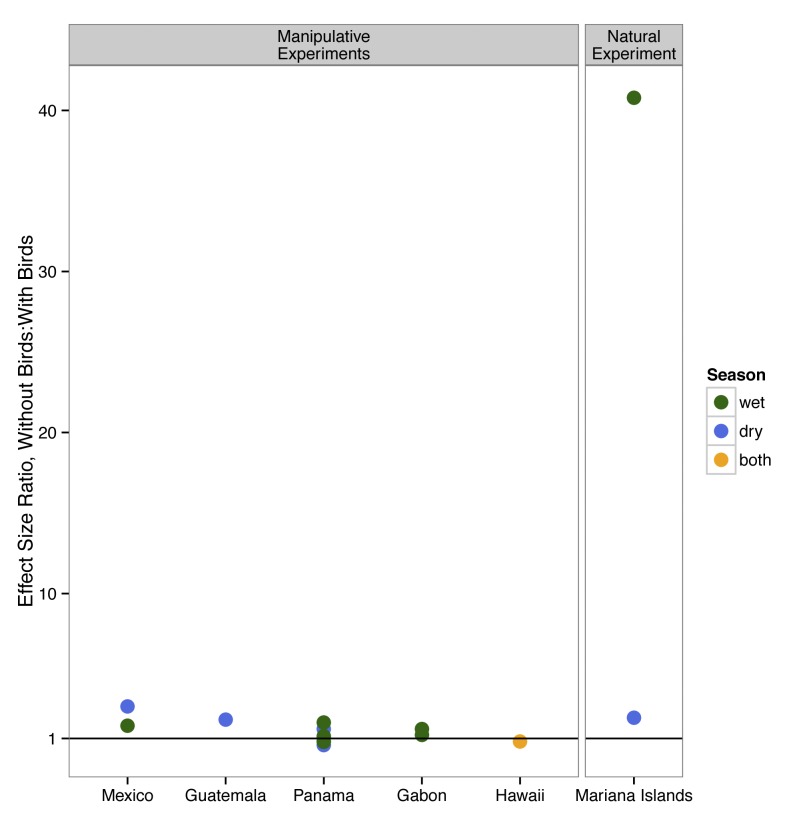


In the Materials and Methods section, under the heading **Insectivorous birds**, the scientific name for the Mariana Swiftlet is listed incorrectly twice in the following sentences:

Only two of the 10 native insectivorous forest bird species remain on Guam: the Micronesian Starling (*Aplonis opaca*), and the Marianas Swiftlet (*Acrocephalus luscinia*). *Aplonis opaca* has a localized remnant population of less than 400 birds (D. Vice, J. Quitagua and L. Obra, pers.comm.) covering an area of less than 50 km2, and *Acrocephalus luscinia* has remnant populations numbering around 1100 in three caves in Southern Guam(A. Brooke, pers.comm.)

The correct name is *Aerodramus bartschi*

